# Refinement of Jensen’s inequality and estimation of *f*- and Rényi divergence via Montgomery identity

**DOI:** 10.1186/s13660-018-1902-9

**Published:** 2018-11-19

**Authors:** Khuram Ali Khan, Tasadduq Niaz, Ðilda Pec̆arić, Josip Pec̆arić

**Affiliations:** 1 Department of Mathematics, University of Sargodha, Sargodha, Pakistan; 2 Department of Mathematics, The University of Lahore, Sargodha, Pakistan; 3 Catholic University of Croatia, Zagreb, Croatia; 4 Rudn University, Moscow, Russia

**Keywords:** *m*-convex function, Jensen’s inequality, *f*- and Rényi divergence, Montgomery identity, Entropy

## Abstract

Jensen’s inequality is important for obtaining inequalities for divergence between probability distribution. By applying a refinement of Jensen’s inequality (Horváth et al. in Math. Inequal. Appl. 14:777–791, 2011) and introducing a new functional based on an *f*-divergence functional, we obtain some estimates for the new functionals, the *f*-divergence, and Rényi divergence. Some inequalities for Rényi and Shannon estimates are constructed. The Zipf–Mandelbrot law is used to illustrate the result. In addition, we generalize the refinement of Jensen’s inequality and new inequalities of Rényi Shannon entropies for an *m*-convex function using the Montgomery identity. It is also given that the maximization of Shannon entropy is a transition from the Zipf–Mandelbrot law to a hybrid Zipf–Mandelbrot law.

## Introduction and preliminary results

The most commonly used words, the largest cities of countries, income of a billionaire can be described in terms of Zipf’s law. The *f*-divergence means the distance between two probability distributions by making an average value, which is weighted by a specified function. As *f*-divergence, there are other probability distributions like Csiszar *f*-divergence [12, 13], some special case of which are Kullback–Leibler-divergence used to find the appropriate distance between the probability distributions (see [20, 21]). The notion of distance is stronger than that of divergence because it gives the properties of symmetry and triangle inequalities. Probability theory has applications in many fields, and the divergence between probability distributions has many applications in these fields.

Many natural phenomena, like distribution of wealth and income in a society, distribution of Facebook likes, distribution of football goals, follow the power law distribution (Zipf’s law). Like above phenomena, the distribution of city sizes also follows the power law distribution. In [4] Auerbach was the first who gave the idea that the distribution of city sizes can be well approximated by using the Pareto distribution (power law distribution). Many researchers refined this idea. However, Zipf [29] has done notable work in this field. Rosen and Resnick [27], Black and Henderson [5], Ioannides and Overman [19], Soo [28], Anderson and Ge [3], and Bosker et al. [6] investigated the distribution of city sizes of the urban economics. It means that the product of the rank appears and city sizes are roughly constant. This shows that population of the *n*th city is $\frac{1}{n}$ of the largest city population. This rule is named rank, size rule and is also called Zipf’s law. Hence Zipf’s law does not only show that the city size distribution follows the Pareto distribution.

By using an *f*-divergence functional, Horváth et al. in [17] introduced a new functional and obtained some estimates for that functional, the Rényi divergence, and *f*-divergence applying a cyclic refinement of Jensen’s inequality. Also they obtained some new inequalities for Shannon and Rényi entropies; also they used the Zipf–Mandelbrot law to illustrate some results.

The inequalities involving higher order convexity are used by many physicists in higher dimension problems since the founding of higher order convexity by Popoviciu (see [25, p. 15]). It is quite an interesting fact that there are some results that are true for convex functions, but when we discuss them in higher order convexity, they do not remain valid.

In [25, p. 16], the following criterion is given to check the *m*-convexity of the function:

If $f^{(m)}$ exists, then *f* is *m*-convex if and only if $f^{(m)} \ge 0$.

In recent years many researchers have generalized the inequalities for *m*-convex functions; for example, Butt et al. generalized the Popoviciu inequality for an *m*-convex function using Taylor’s formula, Lidstone polynomial, Montgomery identity, Fink’s identity, Abel–Gonstcharoff interpolation, and Hermite interpolating polynomial (see [7–11]).

Since many years Jensen’s inequality has received great interest. The researchers have given the refinement of Jensen’s inequality by defining some new functions (see [16, 18]). Like many researchers Horváth and Pec̆arić in [14, 18] (see also [15, p. 26]) gave a refinement of Jensen’s inequality for convex functions. They defined some essential notions to prove the refinement given as follows:

Suppose *X* to be a set, $P(X)$ denotes the power set of *X*, $\vert X\vert $ denotes the number of elements of *X*, and N denotes the nonnegative integers.

Consider $q \ge 1$ and $r \ge 2$ to be fixed integers. Define the functions

$$\begin{aligned}& F_{r,s}:\{ 1, \ldots,q\}^{r} \to \{ 1, \ldots,q \}^{r - 1},\quad 1 \le s \le r, \\& F_{r}:\{ 1, \ldots,q\}^{r} \to P \bigl( \{ 1, \ldots,q \}^{r - 1} \bigr), \end{aligned}$$

and

$$ T_{r}:P \bigl( \{ 1, \ldots,q\}^{r} \bigr) \to P \bigl( \{ 1, \ldots,q\}^{r - 1} \bigr), $$

by

$$\begin{aligned}& F_{r,s}(i_{1}, \ldots,i_{r}): = (i_{1},i_{2}, \ldots,i_{s - 1},i _{s + 1}, \ldots,i_{r}), \quad 1 \le s \le r, \\& F_{r}(i_{1}, \ldots,i_{r}): = \bigcup _{s = 1}^{r} \bigl\{ F_{r,s}(i_{1}, \ldots,i_{r}) \bigr\} , \end{aligned}$$

and

$$ T_{r}(I) = \textstyle\begin{cases} \phi, & I = \phi; \\ \bigcup_{(i_{1}, \ldots,i_{r}) \in I} F_{r}(i_{1}, \ldots,i_{r}), & I \ne \phi. \end{cases} $$

Next let the function

$$ \alpha_{r,i}:\{ 1, \ldots,q\}^{r} \to \mathsf{N}, \quad 1 \le i \le q, $$

be defined by

$$ \alpha_{r,i}(i_{1}, \ldots,i_{r})\quad \text{is the number of occurences of in the sequence }(i_{1}, \ldots,i_{r}). $$

For each $I \in P(\{ 1, \ldots,q\}^{r})$, let

$$ \alpha_{I,i}: = \sum_{(i_{1}, \ldots,i_{r}) \in I} \alpha_{r,i}(i _{1}, \ldots,i_{r}),\quad 1 \le i \le q. $$

($H_{1}$)
Let *n*, *m* be fixed positive integers such that $n \ge 1$, $m \ge 2$, and let $I_{m}$ be a subset of $\{ 1, \ldots,n\}^{m}$ such that
  $$ \alpha_{I_{m},i} \ge 1, \quad 1 \le i \le n. $$

Introduce the sets $I_{l} \subset \{ 1, \ldots,n\}^{l}$ ($m - 1 \ge l \ge 1$) inductively by

$$ I_{l - 1}: = T_{l}(I_{l}),\quad m \ge l \ge 2. $$

Obviously, the sets $I_{1} = \{ 1, \ldots,n\}$ by ($H_{1}$) and this ensures that $\alpha_{I_{1},i} = 1$ ($1 \le i \le n$). From ($H_{1}$) we have $\alpha_{I_{l},i} \ge 1$ ($m - 1 \ge l \ge 1$, $1 \le i \le n$).

For $m \ge l \ge 2$, and for any $(j_{1}, \ldots,j_{l - 1}) \in I_{l - 1}$, let

$$ \mathsf{H}_{I_{l}}(j_{1}, \ldots,j_{l - 1}): = \bigl\{ \bigl((i_{1}, \ldots,i _{l}),k \bigr) \times \{ 1, \ldots,l\} |F_{l,k}(i_{1}, \ldots,i_{l}) = (j _{1}, \ldots,j_{l - 1}) \bigr\} . $$

With the help of these sets, they define the functions $\eta_{I_{m},l}:I_{l} \to \mathsf{N}$ ($m \ge l \ge 1$) inductively by

$$\begin{aligned}& \eta_{I_{m},m}(i_{1}, \ldots,i_{m}): = 1,\quad (i_{1}, \ldots,i_{m}) \in I_{m}; \\& \eta_{I_{m},l - 1}(j_{1}, \ldots,j_{l - 1}): = \sum _{((i_{1}, \ldots,i_{l}),k) \in \mathsf{H}_{I_{l}}(j_{1}, \ldots,j _{l - 1})} \eta_{I_{m},l}(i_{1}, \ldots,i_{l}). \end{aligned}$$

They define some special expressions for $1 \le l \le m$ as follows:

$$\begin{aligned}& \mathsf{A}_{m,l} = \mathsf{A}_{m,l}(I_{m},x_{1}, \ldots,x_{n},p_{1}, \ldots,p_{n};f): = \frac{(m - 1)!}{(l - 1)!} \sum_{(i_{1}, \ldots,i_{l}) \in I_{l}} \eta_{I_{m},l}(i_{1}, \ldots,i_{l}), \\& \Biggl( \sum_{j = 1}^{l} \frac{p_{i_{j}}}{\alpha_{I_{m},i_{j}}} \Biggr) f \biggl( \frac{\sum_{j = 1}^{l} \frac{p_{i_{j}}}{\alpha_{I_{m},i_{j}}}x _{i_{j}}}{\sum_{j = 1}^{l} \frac{p_{i_{j}}}{\alpha_{I_{m},i_{j}}}} \biggr) \end{aligned}$$

and prove the following theorem.

### Theorem 1.1

*Assume* ($H_{1}$), *and let* $f:I \to \mathsf{R}$ *be a convex function where* $I \subset \mathsf{R}$ *is an interval*. *If* $x_{1}, \ldots,x_{n} \in I$ *and* $p_{1}, \ldots,p_{n}$ *are positive real numbers such that* $\sum_{i = 1}^{n} p_{i} = 1$, *then*

1 $$ f \Biggl( \sum_{s = 1}^{n} p_{s}x_{s} \Biggr) \le \mathsf{A}_{m,m} \le \mathsf{A}_{m,m - 1} \le \cdots \le \mathsf{A}_{m,2} \le \mathsf{A}_{m,1} = \sum _{s = 1}^{n} p_{s}f ( x_{s} ). $$

By using the differences of inequalities in (1), we define some new functionals as follows:

2 $$\begin{aligned}& \varTheta_{1}(f) = \mathsf{A}_{m,r} - f \Biggl( \sum _{s = 1}^{n} p_{s}x_{s} \Biggr), \quad r = 1, \ldots,m, \end{aligned}$$

3 $$\begin{aligned}& \varTheta_{2}(f) = \mathsf{A}_{m,r} - \mathsf{A}_{m,k},\quad 1 \le r < k \le m. \end{aligned}$$

Under the assumptions of Theorem 1.1, we have

4 $$ \varTheta_{i}(f) \ge 0,\quad i = 1,2. $$

Inequalities (4) are reversed if *f* is concave on *I*.

The Montgomery identity via Taylor’s formula is given in [1] and [2].

### Theorem 1

*Let* $m \in \mathsf{N}$, $f:I \to \mathsf{R}$ *be such that* $f^{(m - 1)}$ *is absolutely continuous*, $I \subset \mathsf{R}$ *be an open interval*, $\alpha_{1},\alpha _{2} \in I$, $\alpha_{1} < \alpha_{2}$. *Then the following identity holds*:

5 $$\begin{aligned} \psi (x) =& \frac{1}{\alpha_{2} - \alpha_{1}} \int_{\alpha_{1}}^{\alpha _{2}} \psi (u)\,du + \sum _{k = 0}^{m - 2} \frac{\psi^{(k + 1)}(\alpha _{1})(x - \alpha_{1})^{k + 2}}{k!(k + 2)(\alpha_{2} - \alpha_{1})} - \sum _{k = 0}^{m - 2} \frac{\psi^{(k + 1)}(\alpha_{2})(x - \alpha_{2})^{k + 2}}{k!(k + 2)(\alpha_{2} - \alpha_{1})} \\ &{}+ \frac{1}{(m - 1)!} \int_{\alpha_{1}}^{\alpha_{2}} R_{m}(x,u) \psi^{(m)}(u)\,du, \end{aligned}$$

*where*

6 $$ R_{m}(x,u) =\textstyle\begin{cases} - \frac{(x - u)^{m}}{m(\alpha_{2} - \alpha_{1})} + \frac{x - \alpha _{1}}{\alpha_{2} - \alpha_{1}}(x - u)^{m - 1}, & \alpha_{1} \le u \le x; \\ - \frac{(x - u)^{m}}{m(\alpha_{2} - \alpha_{1})} + \frac{x - \alpha _{2}}{\alpha_{2} - \alpha_{1}}(x - u)^{m - 1}, & x \le u \le \alpha_{2}. \end{cases} $$

### Theorem 2

*Let* $m \in \mathsf{N}$, $f:I \to \mathsf{R}$ *be such that* $f^{(m - 1)}$ *is absolutely continuous*, $I \subset \mathsf{R}$ *be an interval*, $\alpha_{1},\alpha_{2} \in I$, $\alpha_{1} < \alpha_{2}$. *Then the following identity holds*:

7 $$\begin{aligned} \psi (x) =& \frac{1}{\alpha_{2} - \alpha_{1}} \int_{\alpha_{1}}^{\alpha _{2}} \psi (u)\,du + \sum _{k = 0}^{m - 2} \psi^{(k + 1)}(x)\frac{(\alpha _{1} - x)^{k + 2} - (\alpha_{2} - x)^{k + 2}}{(k + 2)!(\alpha_{2} - \alpha_{1})} \\ &{} + \frac{1}{(m - 1)!} \int_{\alpha_{1}}^{\alpha_{2}} \hat{R} (x,u) \psi^{(m)}(u) \,du, \end{aligned}$$

*where*

8 $$ \hat{R} (x,u) = \textstyle\begin{cases} - \frac{1}{m(\alpha_{2} - \alpha_{1})}(\alpha_{1} - u), & \alpha_{1} \le u \le x; \\ - \frac{1}{m(\alpha_{2} - \alpha_{1})}(\alpha_{2} - u), & x \le u \le \alpha_{2}. \end{cases} $$

In case $m = 1$, the sum $\sum_{k = 0}^{m - 2} \ldots $ is empty, so (5) and (7) reduce to the well-known Montgomery identity (see [24])

$$ f(x) = \frac{1}{\alpha_{2} - \alpha_{1}} \int_{\alpha_{1}}^{\alpha_{2}} f(t)\,dt + \frac{1}{\alpha_{2} - \alpha_{1}} \int_{\alpha_{1}}^{\alpha _{2}} p(x,u)f'(u)\,du, $$

where $p(x,u)$ is the Peano kernel defined by

$$ p(x,u) = \textstyle\begin{cases} \frac{u - \alpha_{1}}{\alpha_{2} - \alpha_{1}}, & \alpha_{1} \le u \le x; \\ \frac{u - \alpha_{2}}{\alpha_{2} - \alpha_{1}}, & x \le u \le \alpha_{2}. \end{cases} $$

## Inequalities for Csiszár divergence

In [12, 13] Csiszár introduced the following notion.

### Definition 1

Let $f:\mathsf{R}^{ +} \to \mathsf{R}^{ +} $ be a convex function, let $\mathbf{r} = ( r_{1}, \ldots,r_{n} ) $ and $\mathbf{q} = ( q_{1}, \ldots,q_{n} ) $ be positive probability distributions. Then the *f*-divergence functional is defined by

9 $$ I_{f}(\mathbf{r},\mathbf{q}): = \sum_{i = 1}^{n} q_{i}f \biggl( \frac{r_{i}}{q_{i}} \biggr). $$

And he stated that by defining

10 $$ f(0): = \lim_{x \to 0^{ +}} f(x); \qquad 0f \biggl( \frac{0}{0} \biggr): = 0; \qquad 0f \biggl( \frac{a}{0} \biggr): = \lim _{x \to 0^{ +}} xf \biggl( \frac{a}{0} \biggr),\quad a > 0, $$

we can also use the nonnegative probability distributions.

In [17], Horv́ath et al. gave the following functional on the basis of previous definition.

### Definition 2

Let $I \subset \mathsf{R}$ be an interval, and let $f:I \to \mathsf{R}$ be a function, let $\mathbf{r} = (r_{1}, \ldots,r_{n}) \in \mathsf{R}^{n}$ and $\mathbf{q} = (q_{1}, \ldots,q_{n}) \in (0,\infty)^{n}$ such that

$$ \frac{r_{s}}{q_{s}} \in I,\quad s = 1, \ldots,n. $$

Then we define the sum as $\hat{I}_{f}(\mathbf{r},\mathbf{q})$ as

11 $$ \hat{I}_{f}(\mathbf{r},\mathbf{q}): = \sum _{s = 1}^{n} q_{s}f \biggl( \frac{r_{s}}{q_{s}} \biggr). $$

We apply Theorem 1.1 to $\hat{I}_{f}(\mathbf{r},\mathbf{q})$.

### Theorem 2.1

*Assume* ($H_{1}$), *let* $I \subset \mathsf{R}$ *be an interval*, *and let* $\mathbf{r} = ( r_{1}, \ldots,r_{n} ) $ *and* $\mathbf{q} = ( q_{1}, \ldots,q_{n} ) $ *be in* $(0,\infty)^{n}$ *such that*

$$ \frac{r_{s}}{q_{s}} \in I,\quad s = 1, \ldots,n. $$

(i) *If* $f:I \to \mathsf{R}$ *is a convex function*, *then*
  12 $$\begin{aligned} \hat{I}_{f}(\mathbf{r},\mathbf{q}) =& \sum _{s = 1}^{n} q_{s}f \biggl( \frac{r_{s}}{q_{s}} \biggr) = A_{m,1}^{[1]}\ge A_{m,2}^{[1]} \ge \cdots \ge A_{m,m - 1}^{[1]} \\ \ge& A_{m,m}^{[1]} \ge f \biggl( \frac{ \sum_{s = 1}^{n} r_{s}}{\sum_{s = 1}^{n} q_{s}} \biggr) \sum_{s = 1} ^{n} q_{s}, \end{aligned}$$
  *where*
  13 $$ A_{m,l}^{[1]} = \frac{(m - 1)!}{(l - 1)!} \sum _{(i_{1}, \ldots,i_{l}) \in I_{l}} \eta_{I_{m},l}(i_{1}, \ldots,i_{l}) \Biggl( \sum_{j = 1}^{l} \frac{q_{i_{j}}}{\alpha_{I_{m},i_{j}}} \Biggr) f \biggl( \frac{\sum_{j = 1}^{l} \frac{r_{i_{j}}}{ \alpha_{I_{m},i_{j}}}}{\sum_{j = 1}^{l} \frac{q_{i_{j}}}{ \alpha_{I_{m},i_{j}}}} \biggr) . $$
  *If* *f* *is a concave function*, *then the inequality signs in* (12) *are reversed*.
(ii) *If* $f:I \to \mathsf{R}$ *is a function such that* $x \to xf(x)$ ($x \in I$) *is convex*, *then*
  14 $$\begin{aligned} \Biggl( \sum_{s = 1}^{n} r_{s} \Biggr) f \Biggl( \sum_{s = 1}^{n} \frac{r _{s}}{\sum_{s = 1}^{n} q_{s}} \Biggr) \le& A_{m,m}^{[2]} \le A_{m,m - 1} ^{[2]} \le \cdots \le A_{m,2}^{[2]} \le A_{m,1}^{[2]} \\ =& \sum_{s = 1} ^{n} r_{s}f \biggl( \frac{r_{s}}{q_{S}} \biggr) = \hat{I}_{\operatorname{id}f}( \mathbf{r}, \mathbf{q}) , \end{aligned}$$
  *where*
  $$ A_{m,l}^{[2]} = \frac{(m - 1)!}{(l - 1)!} \sum _{(i_{1}, \ldots,i_{l}) \in I_{l}} \eta_{I_{m},l}(i_{1}, \ldots,i_{l}) \Biggl( \sum_{j = 1}^{l} \frac{q_{i_{j}}}{\alpha_{I_{m},i_{j}}} \Biggr) \biggl( \frac{\sum_{j = 1}^{l} \frac{r_{i_{j}}}{\alpha_{I_{m},i _{j}}}}{\sum_{j = 1}^{l} \frac{q_{i_{j}}}{\alpha_{I_{m},i_{j}}}} \biggr) f \biggl( \frac{\sum_{j = 1}^{l} \frac{r_{i_{j}}}{\alpha_{I_{m},i_{j}}}}{ \sum_{j = 1}^{l} \frac{q_{i_{j}}}{\alpha_{I_{m},i_{j}}}} \biggr). $$

### Proof

(i) Considering $p_{s} = \frac{q_{s}}{\sum_{s = 1} ^{n} q_{s}}$ and $x_{s} = \frac{r_{s}}{q_{s}}$ in Theorem 1.1, we have

15 $$\begin{aligned}& \begin{aligned}&f \Biggl( \sum_{s = 1}^{n} \frac{q_{s}}{\sum_{s = 1}^{n} q_{s}}\frac{r _{s}}{q_{s}} \Biggr) \le \cdots \le \frac{(m - 1)!}{(l - 1)!} \sum_{(i_{1}, \ldots,i_{l}) \in I_{l}} \eta_{I_{m},l}(i_{1}, \ldots,i_{l}), \\ & \Biggl( \sum_{j = 1}^{l} \frac{\frac{q_{i_{j}}}{\sum_{s = 1}^{n} q_{s}}}{ \alpha_{I_{m},i_{j}}} \Biggr) f \biggl( \frac{\sum_{j = 1}^{l} \frac{\frac{q _{i_{j}}}{\sum_{i = 1}^{n} q_{i}}}{\alpha_{I_{m},i_{j}}}\frac{r_{i _{j}}}{q_{i_{j}}}}{\sum_{j = 1}^{l} \frac{\frac{q_{i_{j}}}{\sum_{i = 1} ^{n} q_{i}}}{\alpha_{I_{m},i_{j}}}} \biggr) \le \cdots \le \sum _{s = 1} ^{n} \frac{q_{s}}{\sum_{i = 1}^{n} q_{s}}f \biggl( \frac{r_{s}}{q_{s}} \biggr). \end{aligned} \end{aligned}$$

And taking the sum $\sum_{s = 1}^{n} q_{i}$, we have (12).

(ii) Using $f: = \operatorname{id}f$ (where “*id*” is the identity function) in Theorem 1.1, we have

16 $$\begin{aligned}& \begin{aligned}&\sum_{s = 1}^{n} p_{s}x_{s}f \Biggl( \sum_{s = 1}^{n} p_{s}x_{s} \Biggr) \le \cdots \le \frac{(m - 1)!}{(l - 1)!} \sum _{(i_{1}, \ldots,i_{l}) \in I_{l}} \eta_{I_{m},l}(i_{1}, \ldots,i_{l}), \\ & \Biggl( \sum_{j = 1}^{l} \frac{p_{i_{j}}}{\alpha_{I_{m},i_{j}}} \Biggr) \biggl( \frac{\sum_{j = 1}^{l} \frac{p_{i_{j}}}{\alpha_{I_{m},i_{j}}}x _{i_{j}}}{\sum_{j = 1}^{l} \frac{p_{i_{j}}}{\alpha_{I_{m},i_{j}}}} \biggr) f \biggl( \frac{\sum_{j = 1}^{l} \frac{p_{i_{j}}}{\alpha_{I_{m},i_{j}}}x _{i_{j}}}{\sum_{j = 1}^{l} \frac{p_{i_{j}}}{\alpha_{I_{m},i_{j}}}} \biggr) \le \cdots \le \sum_{s = 1}^{n} p_{s}x_{s}f(x_{s}). \end{aligned} \end{aligned}$$

Now, on using $p_{s} = \frac{q_{s}}{\sum_{s = 1}^{n} q_{s}}$ and $x_{s} = \frac{r_{s}}{q_{s}}$, $s = 1, \ldots,n$, we get

17 $$\begin{aligned}& \begin{aligned}&\sum_{s = 1}^{n} \frac{q_{s}}{\sum_{s = 1}^{n} q_{s}}\frac{r_{s}}{q _{s}}f \Biggl( \sum_{s = 1}^{n} \frac{q_{s}}{\sum_{s = 1}^{n} q_{s}}\frac{r _{s}}{q_{s}} \Biggr) \le \cdots \le \frac{(m - 1)!}{(l - 1)!} \sum_{(i_{1}, \ldots,i_{l}) \in I_{l}} \eta_{I_{m},l}(i_{1}, \ldots,i_{l}), \\ & \Biggl( \sum_{j = 1}^{l} \frac{\frac{q_{i_{j}}}{\sum_{s = 1}^{n} q_{s}}}{ \alpha_{I_{m},i_{j}}} \Biggr) \biggl( \frac{\sum_{j = 1}^{l} \frac{\frac{q _{i_{j}}}{\sum_{s = 1}^{n} q_{s}}}{\alpha_{I_{m},i_{j}}}\frac{r_{i _{j}}}{q_{i_{j}}}}{\sum_{j = 1}^{l} \frac{\frac{q_{i_{j}}}{\sum_{s = 1} ^{n} q_{s}}}{\alpha_{I_{m},i_{j}}}} \biggr) f \biggl( \frac{\sum_{j = 1} ^{l} \frac{\frac{q_{i_{j}}}{\sum_{s = 1}^{n} q_{s}}}{\alpha_{I_{m},i _{j}}}\frac{r_{i_{j}}}{q_{i_{j}}}}{\sum_{j = 1}^{l} \frac{\frac{q_{i _{j}}}{\sum_{s = 1}^{n} q_{s}}}{\alpha_{I_{m},i_{j}}}} \biggr) \\ &\quad \le \cdots \le \sum_{s = 1}^{n} \frac{q_{s}}{\sum_{s = 1}^{n} q_{s}}\frac{r _{s}}{q_{s}}f \biggl( \frac{r_{s}}{q_{S}} \biggr). \end{aligned} \end{aligned}$$

On taking sum $\sum_{s = 1}^{n} q_{s}$ on both sides, we get (14). □

## Inequalities for Shannon entropy

### Definition 3

(See [17])

Let $\mathbf{r} = (r_{1}, \ldots,r_{n})$ be a positive probability distribution, the Shannon entropy of **r** is defined by

18 $$ S: = - \sum_{s = 1}^{n} r_{s}\log (r_{s}). $$

### Corollary 3.1

*Assume* ($H_{1}$).

(i) *If* $\mathbf{q} = (q_{1}, \ldots,q_{n}) \in (0,\infty)^{n}$, *and suppose that the base of* log *is greater than* 1, *then*
  19 $$ S \le A_{m,m}^{[3]} \le A_{m,m - 1}^{[3]} \le \cdots \le A_{m,2}^{[3]} \le A_{m,1}^{[3]} = \log \biggl( \frac{n}{\sum_{s = 1}^{n} q_{s}} \biggr) \sum_{s = 1}^{n} q_{s}, $$
  *where*
  20 $$ A_{m,l}^{[3]} = - \frac{(m - 1)!}{(l - 1)!} \sum _{(i_{1}, \ldots,i_{l}) \in I_{l}} \eta_{I_{m},l}(i_{1}, \ldots,i_{l}) \Biggl( \sum_{j = 1}^{l} \frac{q_{i_{j}}}{\alpha_{I_{m},i_{j}}} \Biggr) \log \Biggl( \sum_{j = 1}^{l} \frac{q_{i_{j}}}{\alpha_{I_{m},i _{j}}} \Biggr). $$
  *And in case* log *is between* 0 *and* 1, *then the reverse sign of inequalities holds in* (19).
(ii) *Suppose that the base of* log *is greater than* 1, *if* $\mathbf{q} = (q_{1}, \ldots,q_{n})$ *is a positive probability distribution*, *then*
  21 $$ S \le A_{m,m}^{[4]} \le A_{m,m - 1}^{[4]} \le \cdots \le A_{m,2}^{[4]} \le A_{m,1}^{[4]} = \log (n), $$
  *where*
  $$ A_{m,l}^{[4]} = - \frac{(m - 1)!}{(l - 1)!} \sum _{(i_{1}, \ldots,i_{l}) \in I_{l}} \eta_{I_{m},l}(i_{1}, \ldots,i_{l}) \Biggl( \sum_{j = 1}^{l} \frac{q_{i_{j}}}{\alpha_{I_{m},i_{j}}} \Biggr) \log \Biggl( \sum_{j = 1}^{l} \frac{q_{i_{j}}}{\alpha_{I_{m},i _{j}}} \Biggr). $$

### Proof

(i) Using $f: = \log $ and $\mathbf{r} = (1, \ldots,1)$ in Theorem 2.1(i), we get (19).
(ii) It is the special case of (i).

 □

### Definition 4

(See [17])

Let $\mathbf{r} = (r _{1}, \ldots,r_{n})$ and $\mathbf{q} = (q_{1}, \ldots,q _{n})$ be positive probability distributions, the Kullback–Leibler divergence between **r** and **q** is defined by

22 $$ D(\mathbf{r},\mathbf{q}): = \sum_{s = 1}^{n} r_{i}\log \biggl( \frac{r _{i}}{q_{i}} \biggr). $$

### Corollary 3.2

*Assume* ($H_{1}$).

(i) *Let* $\mathbf{r} = (r_{1}, \ldots,r_{n}) \in (0,\infty)^{n}$ *and* $\mathbf{q}: = (q_{1}, \ldots,q_{n}) \in (0,\infty)^{n}$. *If the base of* log *is greater than* 1, *then*
  23 $$\begin{aligned} \sum_{s = 1}^{n} r_{s} \log \Biggl( \sum_{s = 1}^{n} \frac{r_{s}}{\sum_{s = 1}^{n} q_{s}} \Biggr) \le& A_{m,m}^{[5]} \le A_{m,m - 1}^{[5]} \le \cdots \le A_{m,2}^{[5]} \le A_{m,1}^{[5]} \\ =& \sum_{s = 1}^{n} r _{s}\log \biggl( \frac{r_{s}}{q_{s}} \biggr) = D(\mathbf{r}, \mathbf{q}), \end{aligned}$$
  *where*
  $$ A_{m,l}^{[5]} = \frac{(m - 1)!}{(l - 1)!} \sum _{(i_{1}, \ldots,i_{l}) \in I_{l}} \eta_{I_{m},l}(i_{1}, \ldots,i_{l}) \Biggl( \sum_{j = 1}^{l} \frac{q_{i_{j}}}{\alpha_{I_{m},i_{j}}} \Biggr) \biggl( \frac{\sum_{j = 1}^{l} \frac{r_{i_{j}}}{\alpha_{I_{m},i _{j}}}}{\sum_{j = 1}^{l} \frac{q_{i_{j}}}{\alpha_{I_{m},i_{j}}}} \biggr) \log \biggl( \frac{\sum_{j = 1}^{l} \frac{r_{i_{j}}}{\alpha_{I_{m},i_{j}}}}{ \sum_{j = 1}^{l} \frac{q_{i_{j}}}{\alpha_{I_{m},i_{j}}}} \biggr). $$
  *And in case* log *is between* 0 *and* 1, *then the reverse sign of inequalities holds in* (23).
(ii) *Suppose that the base of* log *is greater than* 1, *if* $\mathbf{r} = (r_{1}, \ldots,r_{n})$ *and* $\mathbf{q} = (q_{1}, \ldots,q_{n})$ *are two positive probability distributions*, *then*
  24 $$ D(\mathbf{r},\mathbf{q}) = A_{m,1}^{[6]} \ge A_{m,2}^{[6]} \ge \cdots \ge A_{m,m - 1}^{[6]} \ge A_{m,m}^{[6]} \ge 0, $$
  *where*
  $$ A_{m,l}^{[6]} = \frac{(m - 1)!}{(l - 1)!} \sum _{(i_{1}, \ldots,i_{l}) \in I_{l}} \eta_{I_{m},l}(i_{1}, \ldots,i_{l}) \Biggl( \sum_{j = 1}^{l} \frac{q_{i_{j}}}{\alpha_{I_{m},i_{j}}} \Biggr) \biggl( \frac{\sum_{j = 1}^{l} \frac{r_{i_{j}}}{\alpha_{I_{m},i _{j}}}}{\sum_{j = 1}^{l} \frac{q_{i_{j}}}{\alpha_{I_{m},i_{j}}}} \biggr) \log \biggl( \frac{\sum_{j = 1}^{l} \frac{r_{i_{j}}}{\alpha_{I_{m},i_{j}}}}{ \sum_{j = 1}^{l} \frac{q_{i_{j}}}{\alpha_{I_{m},i_{j}}}} \biggr). $$
  *And in case* log *is between* 0 *and* 1, *then the reverse sign of inequalities holds in* (24).

### Proof

(i) On taking $f: = \log $ in Theorem 2.1(ii), we get (23).
(ii) It is a special case of (i).

 □

## Inequalities for Rényi divergence and entropy

In [26] Rényi divergence and entropy is given as follows.

### Definition 5

Let $\mathbf{r}: = (r_{1}, \ldots,r_{n})$ and $\mathbf{q}: = (q_{1}, \ldots,q_{n})$ be positive probability distributions, and let $\lambda \ge 0$, $\lambda \ne 1$.

- The Rényi divergence of order *λ* is defined by
  25 $$ D_{\lambda } (\mathbf{r},\mathbf{q}): = \frac{1}{\lambda - 1}\log \Biggl( \sum _{i = 1}^{n} q_{i} \biggl( \frac{r_{i}}{q_{i}} \biggr) ^{ \lambda } \Biggr). $$
- The Rényi entropy of order *λ* of *r* is defined by
  26 $$ H_{\lambda } (\mathbf{r}): = \frac{1}{1 - \lambda } \log \Biggl( \sum _{i = 1}^{n} r_{i}^{\lambda } \Biggr). $$

The Rényi divergence (25) and the Rényi entropy (26) can also be extended to nonnegative probability distributions. Note that $\lim_{\lambda \to 1}D_{\lambda } (\mathbf{r},\mathbf{q}) = D( \mathbf{r},\mathbf{q})$ and $\lim_{\lambda \to 1}H_{\lambda } (\mathbf{r}) = S$.

The next two results are given for Rényi divergence.

### Theorem 4.1

*Assume* ($H_{1}$), *let* $\mathbf{r} = (r_{1}, \ldots,r_{n})$ *and* $\mathbf{q} = (q_{1}, \ldots,q_{n})$ *be probability distributions*.

(i) *If* $0 \le \lambda \le \mu $ *such that* $\lambda,\mu \ne 1$, *and the base of* log *is greater than* 1, *then*
  27 $$ D_{\lambda } (\mathbf{r},\mathbf{q}) \le A_{m,m}^{[7]} \le A _{m,m - 1}^{[7]} \le \cdots \le A_{m,2}^{[7]} \le A_{m,1}^{[7]} = D _{\mu } (\mathbf{r},\mathbf{q}), $$
  *where*
  $$\begin{aligned} A_{m,l}^{[7]} =& \frac{1}{\mu - 1}\log \Biggl( \frac{(m - 1)!}{(l - 1)!} \sum_{(i_{1}, \ldots,i_{l}) \in I_{l}} \eta_{I_{m},l}(i_{1}, \ldots,i_{l}) \Biggl( \sum_{j = 1}^{l} \frac{r_{i_{j}}}{\alpha_{I_{m},i_{j}}} \Biggr) \\ &{}\times \biggl( \frac{\sum_{j = 1}^{l} \frac{r_{i_{j}}}{\alpha_{I_{m},i _{j}}} ( \frac{r_{i_{j}}}{q_{i_{j}}} ) ^{\lambda - 1}}{ \sum_{j = 1}^{l} \frac{r_{i_{j}}}{\alpha_{I_{m},i_{j}}}} \biggr) ^{\frac{ \mu - 1}{\lambda - 1}} \Biggr) . \end{aligned}$$
  *And in case* log *is between* 0 *and* 1, *then the reverse sign of inequalities holds in* (27).
(ii) *If the base of* log *is greater than* 1 *and* $\mu > 1$, *then*
  28 $$\begin{aligned} D_{1}(\mathbf{r},\mathbf{q}) =& D(\mathbf{r}, \mathbf{q}) = \sum_{s = 1}^{n} r_{s}\log \biggl( \frac{r_{s}}{q_{s}} \biggr) \le A_{m,m}^{[8]} \le A_{m,m - 1}^{[8]} \le \cdots \le A_{m,2} ^{[8]} \le A_{m,1}^{[8]} \\ =& D_{\mu } (\mathbf{r},\mathbf{q}), \end{aligned}$$
  *where*
  $$\begin{aligned} A_{m,l}^{[8]} =& \frac{1}{\mu - 1}\log \Biggl( \frac{(m - 1)!}{(l - 1)!} \sum_{(i_{1}, \ldots,i_{l}) \in I_{l}} \eta_{I_{m},l}(i_{1}, \ldots,i_{l}) \Biggl( \sum_{j = 1}^{l} \frac{r_{i_{j}}}{\alpha_{I_{m},i_{j}}} \Biggr) \\ &{}\times \operatorname{exp}\biggl( \frac{(\mu - 1)\sum_{j = 1}^{l} \frac{r _{i_{j}}}{\alpha_{I_{m},i_{j}}}\log ( \frac{r_{i_{j}}}{q_{i_{j}}} ) }{ \sum_{j = 1}^{l} \frac{r_{i_{j}}}{\alpha_{I_{m},i_{j}}}} \biggr) \Biggr) . \end{aligned}$$
  *Here*, *the* exp *and* log *functions have the same bases*, *and if the base of* log *is in the interval* $(0,1)$, *then the reverse sign of inequalities holds in* (28).
(iii) *If* $0 \le \lambda < 1$, *and the base of* log *is greater than* 1, *then*
  29 $$ D_{\lambda } (\mathbf{r},\mathbf{q}) \le A_{m,m}^{[9]} \le A _{m,m - 1}^{[9]} \le \cdots \le A_{m,2}^{[9]} \le A_{m,1}^{[9]} = D _{1}(\mathbf{r},\mathbf{q}), $$
  *where*
  30 $$\begin{aligned} A_{m,l}^{[9]} =& \frac{1}{\lambda - 1} \frac{(m - 1)!}{(l - 1)!} \sum_{(i_{1}, \ldots,i_{l}) \in I_{l}} \eta_{I_{m},l}(i_{1}, \ldots,i_{l}) \Biggl( \sum_{j = 1}^{l} \frac{r_{i_{j}}}{\alpha_{I_{m},i_{j}}} \Biggr) \\ &{}\times \log \biggl( \frac{\sum_{j = 1}^{l} \frac{r_{i_{j}}}{ \alpha_{I_{m},i_{j}}} ( \frac{r_{i_{j}}}{q_{i_{j}}} ) ^{ \lambda - 1}}{\sum_{j = 1}^{l} \frac{r_{i_{j}}}{\alpha_{I_{m},i_{j}}}} \biggr). \end{aligned}$$

### Proof

By taking $I = (0,\infty)$, $f:(0,\infty) \to \mathsf{R}$, $f(t): = t^{\frac{\mu - 1}{\lambda - 1}}$

$$ p_{s}: = r_{s}, \qquad x_{s}: = \biggl( \frac{r_{s}}{q_{s}} \biggr) ^{\lambda - 1}, \quad s = 1, \ldots,n, $$

in Theorem 1.1, we have

31 $$\begin{aligned}& \Biggl( \sum_{s = 1}^{n} q_{s} \biggl( \frac{r_{s}}{q_{s}} \biggr) ^{ \lambda } \Biggr) ^{\frac{\mu - 1}{\lambda - 1}} \\& \quad = \Biggl( \sum_{s = 1} ^{n} r_{s} \biggl( \frac{r_{s}}{q_{s}} \biggr) ^{\lambda - 1} \Biggr) ^{\frac{\mu - 1}{\lambda - 1}} \\& \quad \le \ldots \le \frac{(m - 1)!}{(l - 1)!} \sum_{(i_{1}, \ldots,i_{l}) \in I_{l}} \eta_{I_{m},l}(i_{1}, \ldots,i_{l}) \Biggl( \sum _{j = 1}^{l} \frac{r_{i_{j}}}{\alpha_{I_{m},i_{j}}} \Biggr) \biggl( \frac{\sum_{j = 1}^{l} \frac{r_{i_{j}}}{\alpha_{I_{m},i _{j}}} ( \frac{r_{i_{j}}}{q_{i_{j}}} ) ^{\lambda - 1}}{ \sum_{j = 1}^{l} \frac{r_{i_{j}}}{\alpha_{I_{m},i_{j}}}} \biggr) ^{\frac{ \mu - 1}{\lambda - 1}} \\& \quad \le \cdots \le \sum_{s = 1}^{n} r_{s} \biggl( \biggl( \frac{r_{s}}{q_{s}} \biggr) ^{\lambda - 1} \biggr) ^{\frac{\mu - 1}{ \lambda - 1}}, \end{aligned}$$

if either $0 \le \lambda < 1 < \beta $ or $1 < \lambda \le \mu $, and the reverse inequality in (31) holds if $0 \le \lambda \le \beta < 1$. By raising to power $\frac{1}{\mu - 1}$, we have from all

32 $$\begin{aligned}& \Biggl( \sum_{s = 1}^{n} q_{s} \biggl( \frac{r_{s}}{q_{s}} \biggr) ^{ \lambda } \Biggr) ^{\frac{1}{\lambda - 1}} \\& \quad \le\ldots \le \Biggl( \frac{(m - 1)!}{(l - 1)!} \sum_{(i_{1}, \ldots,i_{l}) \in I_{l}} \eta_{I_{m},l}(i_{1}, \ldots,i_{l}) \Biggl( \sum _{j = 1}^{l} \frac{r_{i_{j}}}{\alpha_{I_{m},i_{j}}} \Biggr) \\& \qquad {}\times \biggl( \frac{\sum_{j = 1}^{l} \frac{r_{i_{j}}}{\alpha_{I_{m},i _{j}}} ( \frac{r_{i_{j}}}{q_{i_{j}}} ) ^{\lambda - 1}}{ \sum_{j = 1}^{l} \frac{r_{i_{j}}}{\alpha_{I_{m},i_{j}}}} \biggr) ^{\frac{ \mu - 1}{\lambda - 1}} \Biggr) ^{\frac{1}{\mu - 1}} \\& \quad \le \cdots \le \Biggl( \sum_{s = 1}^{n} r_{s} \biggl( \biggl( \frac{r_{s}}{q _{s}} \biggr) ^{\lambda - 1} \biggr) ^{\frac{\mu - 1}{\lambda - 1}} \Biggr) ^{\frac{1}{\mu - 1}} = \Biggl( \sum _{s = 1}^{n} q_{s} \biggl( \frac{r _{s}}{q_{s}} \biggr) ^{\mu } \Biggr) ^{\frac{1}{\mu - 1}}. \end{aligned}$$

Since the log function is increasing for the base greater than 1, therefore on taking log in (32) we get (29). And the log function is decreasing for the base between 0 and 1, in this case on taking log in (32) we get the reverse sign in (27). If $\lambda = 1$ and $\beta = 1$, we have (ii) and (iii) respectively by taking limit. □

### Theorem 4.2

*Assume* ($H_{1}$), *let* $\mathbf{r} = (r_{1}, \ldots,r_{n})$ *and* $\mathbf{q} = (q_{1}, \ldots,q_{n})$ *be probability distributions*. *If either* $0 \le \lambda < 1$ *and the base of* log *is greater than* 1, *or* $1 < \lambda $ *and the base of* log *is between* 0 *and* 1, *then*

33 $$\begin{aligned} \frac{1}{\sum_{s = 1}^{n} q_{s} ( \frac{r_{s}}{q_{s}} ) ^{ \lambda }} \sum_{s = 1}^{n} q_{s} \biggl( \frac{r_{s}}{q_{s}} \biggr) ^{\lambda } \log \biggl( \frac{r_{s}}{q_{s}} \biggr) = &A_{m,1}^{[10]} \le A_{m,2}^{[10]} \le \cdots \le A_{m,m - 1}^{[10]} \le A_{m,m}^{[10]} \\ \le& D_{\lambda } (r,q) \le A_{m,m}^{[11]} \\ \le& A_{m,m}^{[11]} \le \cdots \le A_{m,2}^{[11]} \le A_{m,1}^{[11]} = D_{1}(\mathbf{r},\mathbf{q}) , \end{aligned}$$

*where*

$$\begin{aligned} A_{m,m}^{[10]} = &\frac{1}{(\lambda - 1)\sum_{s = 1}^{n} q_{s} ( \frac{r_{s}}{q _{s}} ) ^{\lambda }} \frac{(m - 1)!}{(l - 1)!} \sum_{(i_{1}, \ldots,i_{l}) \in I_{l}} \eta_{I_{m},l}(i_{1}, \ldots,i_{l}) \Biggl( \sum_{j = 1}^{l} \frac{r_{i_{j}}}{\alpha_{I_{m},i_{j}}} \biggl( \frac{r_{i_{j}}}{q_{i_{j}}} \biggr) ^{\lambda - 1} \Biggr) \\ &{}\times \log \biggl( \frac{\sum_{j = 1}^{l} \frac{r_{i_{j}}}{\alpha_{I_{m},i_{j}}} ( \frac{r_{i_{j}}}{q_{i_{j}}} ) ^{\lambda - 1}}{\sum_{j = 1} ^{l} \frac{r_{i_{j}}}{\alpha_{I_{m},i_{j}}}} \biggr) \end{aligned}$$

*and*

$$\begin{aligned} A_{m,m}^{[11]} =& \frac{1}{\lambda - 1} \frac{(m - 1)!}{(l - 1)!} \sum_{(i_{1}, \ldots,i_{l}) \in I_{l}} \eta_{I_{m},l}(i_{1}, \ldots,i_{l}) \Biggl( \sum_{j = 1}^{l} \frac{r_{i_{j}}}{\alpha_{I_{m},i_{j}}} \Biggr) \\ &{}\times \log \biggl( \frac{\sum_{j = 1}^{l} \frac{r_{i_{j}}}{ \alpha_{I_{m},i_{j}}} ( \frac{r_{i_{j}}}{q_{i_{j}}} ) ^{ \lambda - 1}}{\sum_{j = 1}^{l} \frac{r_{i_{j}}}{\alpha_{I_{m},i_{j}}}} \biggr). \end{aligned}$$

The inequalities in (33) are reversed if either $0 \le \lambda < 1$ and the base of log is between 0 and 1, or $1 < \lambda $ and the base of log is greater than 1.

### Proof

Here we prove for $0 \le \lambda < 1$ and base when the base of logis greater than 1, the other case can be proved by following similar steps. Since $\frac{1}{\lambda - 1} < 0$ and the function log is concave, then choosing $I = (0,\infty)$, $f: = \log $, $p_{s} = r_{s}$, $x_{s}: = ( \frac{r_{s}}{q_{s}} ) ^{\lambda - 1}$ in Theorem 1.1, we have

34 $$\begin{aligned} D_{\lambda } (\mathbf{r},\mathbf{q}) =& \frac{1}{\lambda - 1}\log \Biggl( \sum _{s = 1}^{n} q_{s} \biggl( \frac{r_{s}}{q_{s}} \biggr) ^{ \lambda } \Biggr) = \frac{1}{\lambda - 1} \log \Biggl( \sum_{s = 1}^{n} r _{s} \biggl( \frac{r_{s}}{q_{s}} \biggr) ^{\lambda - 1} \Biggr) \\ \le& \cdots \le \frac{1}{\lambda - 1}\frac{(m - 1)!}{(l - 1)!} \sum _{(i_{1}, \ldots,i_{l}) \in I_{l}} \eta_{I_{m},l}(i_{1}, \ldots,i_{l}) \Biggl( \sum_{j = 1}^{l} \frac{r_{i_{j}}}{\alpha_{I_{m},i_{j}}} \Biggr) \\ &{}\times \log \biggl( \frac{\sum_{j = 1}^{l} \frac{r_{i_{j}}}{ \alpha_{I_{m},i_{j}}} ( \frac{r_{i_{j}}}{q_{i_{j}}} ) ^{ \lambda - 1}}{\sum_{j = 1}^{l} \frac{r_{i_{j}}}{\alpha_{I_{m},i_{j}}}} \biggr) \\ \le& \cdots \le \frac{1}{\lambda - 1}\sum_{s = 1}^{n} r_{s}\log \biggl( \biggl( \frac{r_{s}}{q_{s}} \biggr) ^{\lambda - 1} \biggr) = \sum_{s = 1} ^{n} r_{s} \log \biggl( \frac{r_{s}}{q_{s}} \biggr) = D_{1}( \mathbf{r}, \mathbf{q}) \end{aligned}$$

and this gives the upper bound for $D_{\lambda } (\mathbf{r},\mathbf{q})$.

Since $x \mapsto x\log (x)$ ($x > 0$) is a convex function for base of loglogloglog greater than 1, also $\frac{1}{1 - \lambda } < 0$, therefore using Theorem 1.1, we have

35 $$\begin{aligned} D_{\lambda } (\mathbf{r},\mathbf{q}) =& \frac{1}{\lambda - 1}\log \Biggl( \sum _{s = 1}^{n} q_{s} \biggl( \frac{r_{s}}{q_{s}} \biggr) ^{ \lambda } \Biggr) \\ =& \frac{1}{\lambda - 1 ( \sum_{s = 1}^{n} q_{s} ( \frac{r_{s}}{q_{s}} ) ^{\lambda } ) } \Biggl( \sum_{s = 1}^{n} q_{s} \biggl( \frac{r_{s}}{q_{s}} \biggr) ^{\lambda } \Biggr) \log \Biggl( \sum_{s = 1}^{n} q_{s} \biggl( \frac{r_{s}}{q_{s}} \biggr) ^{ \lambda } \Biggr) \\ \ge& \cdots \ge \frac{1}{\lambda - 1 ( \sum_{s = 1}^{n} q_{s} ( \frac{r_{s}}{q_{s}} ) ^{\lambda } ) } \frac{(m - 1)!}{(l - 1)!}\sum _{(i_{1}, \ldots,i_{l}) \in I_{l}} \eta_{I_{m},l}(i_{1}, \ldots,i_{l}) \Biggl( \sum_{j = 1}^{l} \frac{r _{i_{j}}}{\alpha_{I_{m},i_{j}}} \Biggr) \\ &{}\times \biggl( \frac{\sum_{j = 1}^{l} \frac{r_{i_{j}}}{\alpha_{I_{m},i_{j}}} ( \frac{r_{i_{j}}}{q_{i_{j}}} ) ^{\lambda - 1}}{\sum_{j = 1} ^{l} \frac{r_{i_{j}}}{\alpha_{I_{m},i_{j}}}} \biggr) \log \biggl( \frac{ \sum_{j = 1}^{l} \frac{r_{i_{j}}}{\alpha_{I_{m},i_{j}}} ( \frac{r _{i_{j}}}{q_{i_{j}}} ) ^{\lambda - 1}}{\sum_{j = 1}^{l} \frac{r _{i_{j}}}{\alpha_{I_{m},i_{j}}}} \biggr) \\ =&\frac{1}{\lambda - 1 ( \sum_{s = 1}^{n} q_{s} ( \frac{r_{s}}{q _{s}} ) ^{\lambda } ) }\frac{(m - 1)!}{(l - 1)!} \sum_{(i_{1}, \ldots,i_{l}) \in I_{l}} \eta_{I_{m},l}(i_{1}, \ldots,i_{l}) \\ &{}\times \Biggl( \sum_{j = 1}^{l} \frac{r_{i_{j}}}{\alpha_{I_{m},i_{j}}} \biggl( \frac{r _{i_{j}}}{q_{i_{j}}} \biggr) ^{\lambda - 1} \Biggr) \log \biggl( \frac{ \sum_{j = 1}^{l} \frac{r_{i_{j}}}{\alpha_{I_{m},i_{j}}} ( \frac{r _{i_{j}}}{q_{i_{j}}} ) ^{\lambda - 1}}{\sum_{j = 1}^{l} \frac{r _{i_{j}}}{\alpha_{I_{m},i_{j}}}} \biggr) \\ \ge& \cdots \\ \ge&\frac{1}{\lambda - 1}\sum_{s = 1}^{n} r_{s} \biggl( \frac{r_{s}}{q_{s}} \biggr) ^{\lambda - 1}\log \biggl( \frac{r_{s}}{q_{s}} \biggr) ^{\lambda - 1}\frac{1}{\sum_{s = 1}^{n} r_{s} ( \frac{r_{s}}{q_{s}} ) ^{\lambda - 1}} \\ =& \frac{1}{\sum_{s = 1}^{n} q_{s} ( \frac{r_{s}}{q _{s}} ) ^{\lambda }} \sum_{s = 1}^{n} q_{s} \biggl( \frac{r_{s}}{q _{s}} \biggr) ^{\lambda } \log \biggl( \frac{r_{s}}{q_{s}} \biggr) \end{aligned}$$

which gives the lower bound of $D_{\lambda } (\mathbf{r},\mathbf{q})$.

By using the previous results, some inequalities of Rényi entropy are obtained. Let $\frac{1}{\mathbf{n}} = (\frac{1}{n}, \ldots,\frac{1}{n})$ be a discrete probability distribution. □

### Corollary 4.3

*Assume* ($H_{1}$), *let* $\mathbf{r} = (r_{1}, \ldots,r_{n})$ *and* $\mathbf{q} = (q_{1}, \ldots,q_{n})$ *be positive probability distributions*.

(i) *If* $0 \le \lambda \le \mu $, $\lambda,\mu \ne 1$, *and the base of* log *is greater than* 1, *then*
  36 $$ H_{\lambda } (\mathbf{r}) = \log (n) - D_{\lambda } \biggl( \mathbf{r},\frac{\mathbf{1}}{\mathbf{n}} \biggr) \ge A _{m,m}^{[12]} \ge A_{m,m}^{[12]} \ge \cdots\ge A_{m,2}^{[12]} \ge A_{m,1} ^{[12]} = H_{\mu } (\mathbf{r}), $$
  *where*
  $$\begin{aligned} A_{m,l}^{[12]} =& \frac{1}{1 - \mu } \log \Biggl( \frac{(m - 1)!}{(l - 1)!}\sum_{(i_{1}, \ldots,i_{l}) \in I_{l}} \eta_{I_{m},l}(i_{1}, \ldots,i_{l}) \\ &{} \times \Biggl( \sum_{j = 1}^{l} \frac{r_{i_{j}}}{\alpha_{I_{m},i_{j}}} \Biggr) \biggl( \frac{\sum_{j = 1}^{l} \frac{r_{i_{j}}^{\lambda }}{\alpha_{I_{m},i _{j}}}}{\sum_{j = 1}^{l} \frac{r_{i_{j}}}{\alpha_{I_{m},i_{j}}}} \biggr) ^{\frac{\mu - 1}{\lambda - 1}} \Biggr). \end{aligned}$$
  *The reverse inequalities hold in* (36) *if the base of* log *is between* 0 *and* 1.
(ii) *If* $1 < \mu $ *and the base of* log *is greater than* 1, *then*
  37 $$ S = - \sum_{s = 1}^{n} p_{i}\log (p_{i}) \ge A_{m,m}^{[13]} \ge A_{m,m - 1}^{[13]} \ge \cdots \ge A_{m,2}^{[13]} \ge A_{m,1}^{[13]} = H_{ \mu } (\mathbf{r}), $$
  *where*
  $$\begin{aligned} A_{m,l}^{[13]} =& \log (n) + \frac{1}{1 - \mu } \log \Biggl( \frac{(m - 1)!}{(l - 1)!}\sum_{(i_{1}, \ldots,i_{l}) \in I_{l}} \eta_{I_{m},l}(i _{1}, \ldots,i_{l}) \Biggl( \sum _{j = 1}^{l} \frac{r_{i_{j}}}{ \alpha_{I_{m},i_{j}}} \Biggr) \\ &{}\times \operatorname{exp}\biggl( \frac{(\mu - 1) \sum_{j = 1}^{l} \frac{r_{i_{j}}}{\alpha_{I_{m},i_{j}}}\log ( nr _{i_{j}} ) }{\sum_{j = 1}^{l} \frac{r_{i_{j}}}{\alpha_{I_{m},i _{j}}}} \biggr) \Biggr), \end{aligned}$$
  *the* exp *and* log *functios have the same bases*. *If the base of* log *is between* 0 *and* 1, *the sign of inequalities in* (37) *is reversed*.
(iii) *If* $0 \le \lambda < 1$ *and the base of* log *is greater than* 1, *then*
  38 $$ H_{\lambda } (\mathbf{r}) \ge A_{m,m}^{[14]} \ge A_{m,m - 1}^{[14]} \ge \cdots \ge A_{m,2}^{[14]} \le A_{m,1}^{[14]} = S, $$
  *where*
  39 $$\begin{aligned} A_{m,m}^{[14]} =& \frac{1}{1 - \lambda } \frac{(m - 1)!}{(l - 1)!} \sum _{(i_{1}, \ldots,i_{l}) \in I_{l}} \eta_{I_{m},l}(i_{1}, \ldots,i_{l}) \Biggl( \sum_{j = 1}^{l} \frac{r_{i_{j}}}{\alpha_{I_{m},i_{j}}} \Biggr) \\ &{}\times \log \biggl( \frac{\sum_{j = 1}^{l} \frac{r_{i_{j}}^{\lambda }}{ \alpha_{I_{m},i_{j}}}}{\sum_{j = 1}^{l} \frac{r_{i_{j}}}{ \alpha_{I_{m},i_{j}}}} \biggr). \end{aligned}$$

*The inequalities in* (38) *are reversed if the base of* log *is between* 0 *and* 1.

### Proof

(i) Suppose $\mathbf{q} = \frac{\mathbf{1}}{\mathbf{n}}$, then from (25) we have

40 $$ D_{\lambda } (\mathbf{r},\mathbf{q}) = \frac{1}{\lambda - 1}\log \Biggl( \sum _{s = 1}^{n} n^{\lambda - 1}r_{s}^{\lambda } \Biggr) = \log (n) + \frac{1}{\lambda - 1}\log \Biggl( \sum _{s = 1}^{n} r_{s}^{\lambda } \Biggr), $$

therefore we have

41 $$ H_{\lambda } (\mathbf{r}) = \log (n) - D_{\lambda } \biggl( \mathbf{r}, \frac{ \mathbf{1}}{\mathbf{n}} \biggr). $$

Now, using Theorem 4.1(i) and (41), we get

42 $$\begin{aligned} H_{\lambda } (\mathbf{r}) =& \log (n) - D_{\lambda } \biggl( \mathbf{r},\frac{\mathbf{1}}{\mathbf{n}} \biggr) \ge \cdots \\ \ge& \log (n) - \frac{1}{\mu - 1}\log \Biggl( n^{\mu - 1} \frac{(m - 1)!}{(l - 1)!}\sum_{(i_{1}, \ldots,i_{l}) \in I_{l}} \eta_{I_{m},l}(i _{1}, \ldots,i_{l}) \\ &{} \times \Biggl( \sum_{j = 1}^{l} \frac{r_{i_{j}}}{ \alpha_{I_{m},i_{j}}} \Biggr) \biggl( \frac{\sum_{j = 1}^{l} \frac{r _{i_{j}}^{\lambda }}{\alpha_{I_{m},i_{j}}}}{\sum_{j = 1}^{l} \frac{r _{i_{j}}}{\alpha_{I_{m},i_{j}}}} \biggr) ^{\frac{\mu - 1}{\lambda - 1}} \Biggr) \ge \cdots \\ \ge& \log (n) - D_{\mu } (\mathbf{r}, \mathbf{q}) = H_{\mu } ( \mathbf{r}), \end{aligned}$$

(ii) and (iii) can be proved similarly. □

### Corollary 4.4

*Assume* ($H_{1}$) *and let* $\mathbf{r} = (r_{1}, \ldots,r_{n})$ *and* $\mathbf{q} = (q_{1}, \ldots,q_{n})$ *be positive probability distributions*.

If either $0 \le \lambda < 1$ and the base of log is greater than 1, or $1 < \lambda $ and the base of log is between 0 and 1, then

43 $$\begin{aligned} - \frac{1}{\sum_{s = 1}^{n} r_{s}^{\lambda }} \sum_{s = 1}^{n} r_{s} ^{\lambda } \log (r_{s}) =& A_{m,1}^{[15]} \ge A_{m,2}^{[15]} \ge \cdots \ge A_{m,m - 1}^{[15]} \ge A_{m,m}^{[15]} \\ \ge& H_{\lambda } (r) \ge A _{m,m}^{[16]} \ge A_{m,m - 1}^{[16]} \ge \cdots\ge A_{m,2}^{[16]} \ge A _{m,1}^{[16]} = H ( r ), \end{aligned}$$

where

$$\begin{aligned} A_{m,l}^{[15]} =& \frac{1}{(\lambda - 1)\sum_{s = 1}^{n} r_{s}^{\lambda }} \frac{(m - 1)!}{(l - 1)!}\sum_{(i_{1}, \ldots,i_{l}) \in I_{l}} \eta_{I_{m},l}(i_{1}, \ldots,i_{l}) \Biggl( \sum_{j = 1}^{l} \frac{r _{i_{j}}^{\lambda }}{\alpha_{I_{m},i_{j}}} \Biggr) \\ &{} \times \log \biggl( n^{\lambda - 1}\frac{\sum_{j = 1}^{l} \frac{r_{i_{j}}^{\lambda }}{ \alpha_{I_{m},i_{j}}}}{\sum_{j = 1}^{l} \frac{r_{i_{j}}}{ \alpha_{I_{m},i_{j}}}} \biggr) \end{aligned}$$

and

$$ A_{m,1}^{[16]} = \frac{1}{1 - \lambda } \frac{(m - 1)!}{(l - 1)!} \sum _{(i_{1}, \ldots,i_{l}) \in I_{l}} \eta_{I_{m},l}(i_{1}, \ldots,i_{l}) \Biggl( \sum_{j = 1}^{l} \frac{r_{i_{j}}}{\alpha_{I_{m},i_{j}}} \Biggr) \log \biggl( \frac{\sum_{j = 1}^{l} \frac{r_{i_{j}}^{\lambda }}{ \alpha_{I_{m},i_{j}}}}{\sum_{j = 1}^{l} \frac{r_{i_{j}}}{ \alpha_{I_{m},i_{j}}}} \biggr). $$

The inequalities in (43) are reversed if either $0 \le \lambda < 1$ and the base of log is between 0 and 1, or $1 < \lambda $ and the base of log is greater than 1.

### Proof

The proof is similar to Corollary 4.3 by using Theorem 4.2. □

## Inequalities by using Zipf–Mandelbrot law

In [22] the Zipf–Mandelbrot law is defined as follows.

### Definition 6

The Zipf–Mandelbrot law is a discrete probability distribution depending on three parameters $q \in [0,\infty)$, $N \in \{ 1,2, \ldots\}$, and $t > 0$, and it is defined by

44 $$ f(s;N,q,t): = \frac{1}{(s + q)^{t}H_{N,q,t}}, \quad s = 1, \ldots,N, $$

where

45 $$ H_{N,q,t} = \sum_{j = 1}^{N} \frac{1}{(j + q)^{t}}. $$

If the total mass of the law is taken over all N, then for $q \ge 0$, $t > 1$, $s \in \mathsf{N}$, the density function of the Zipf–Mandelbrot law becomes

46 $$ f(s;q,t) = \frac{1}{(s + q)^{t}H_{q,t}}, $$

where

47 $$ H_{q,t} = \sum_{j = 1}^{\infty } \frac{1}{(j + q)^{t}}. $$

For $q = 0$, the Zipf–Mandelbrot law becomes Zipf’s law.

### Conclusion 5.1

*Assume* ($H_{1}$), *let* *r* *be a Zipf–Mandelbrot law*, *by Corollary *4.3(*iii*), *we get*: *If* $0 \le \lambda < 1$ *and the base of* log *is greater than* 1, *then*

48 $$\begin{aligned} H_{\lambda } (\mathbf{r}) =& \frac{1}{1 - \lambda } \log \Biggl( \frac{1}{H _{N,q,t}^{\lambda }} \sum_{s = 1}^{n} \frac{1}{(s + q)^{\lambda s}} \Biggr) \ge \cdots \\ \ge&\frac{1}{1 - \lambda } \frac{(m - 1)!}{(l - 1)!} \sum_{(i_{1}, \ldots,i_{l}) \in I_{l}} \eta_{I_{m},l}(i_{1}, \ldots,i_{l}) \Biggl( \sum _{j = 1}^{l} \frac{1}{\alpha_{I_{m},i_{j}}(i_{j} + q)H _{N,q,t}} \Biggr) \\ &{}\times \log \biggl( \frac{1}{H_{N,q,t}^{\lambda - 1}}\frac{ \sum_{j = 1}^{l} \frac{1}{\alpha_{I_{m},i_{j}}(i_{j} - q)^{\lambda s}}}{\sum_{j = 1} ^{l} \frac{1}{\alpha_{I_{m},i_{j}}(i_{j} - q)^{s}}} \biggr) \ge \cdots \\ \ge&\frac{t}{H_{N,q,t}}\sum_{s = 1}^{N} \frac{\log (s + q)}{(s + q)^{t}} + \log (H _{N,q,t}) = S. \end{aligned}$$

The inequalities in (48) are reversed if the base of log is between 0 and 1.

### Conclusion 5.2

*Assume* ($H_{1}$), *let* $r_{1}$ *and* $r_{2}$ *be the Zipf–Mandelbort law with parameters* $N \in \{ 1,2, \ldots \}$, $q_{1},q_{2} \in [0,\infty )$, *and* $s_{1},s_{2} > 0$, *respectively*. *Then from Corollary *3.2(*ii*), *we have*: *If the base of* log *is greater than* 1, *then*

49 $$\begin{aligned}& \begin{aligned}&\bar{D}(r_{1},r_{2}) = \sum _{s = 1}^{n} \frac{1}{(s + q_{1})^{t_{1}}H _{N,q_{1},t_{1}}}\log \biggl( \frac{(s + q_{2})^{t_{2}}H_{N,q_{2},t_{2}}}{(s + q_{1})^{t_{1}}H_{N,q_{2},t_{1}}} \biggr) \ge \cdots \\ &\hphantom{\bar{D}(r_{1},r_{2})}\ge \frac{(m - 1)!}{(l - 1)!} \sum_{(i_{1}, \ldots,i_{l}) \in I_{l}} \eta_{I_{m},l}(i_{1}, \ldots,i_{l}), \\ & \Biggl( \sum_{j = 1}^{l} \frac{\frac{1}{(i_{j} + q_{2})^{t_{2}}H_{N,q _{2},t_{2}}}}{\alpha_{I_{m},i_{j}}} \Biggr) \biggl( \frac{\sum_{j = 1} ^{l} \frac{\frac{1}{(i_{j} + q_{1})^{t_{1}}H_{N,q_{1},t_{1}}}}{ \alpha_{I_{m},i_{j}}}}{\sum_{j = 1}^{l} \frac{\frac{1}{(i_{j} + q_{2})^{t _{2}}H_{N,q_{2},t_{2}}}}{\alpha_{I_{m},i_{j}}}} \biggr) \log \biggl( \frac{ \sum_{j = 1}^{l} \frac{\frac{1}{(i_{j} + q_{1})^{t_{1}}H_{N,q_{1},t _{1}}}}{\alpha_{I_{m},i_{j}}}}{\sum_{j = 1}^{l} \frac{\frac{1}{(i_{j} + q_{2})^{t_{2}}H_{N,q_{2},t_{2}}}}{\alpha_{I_{m},i_{j}}}} \biggr) \\ &\quad \ge \cdots \ge 0. \end{aligned} \end{aligned}$$

The inequalities in (49) are reversed if the base of log is between 0 and 1.

## Shannon entropy, Zipf–Mandelbrot law and hybrid Zipf–Mandelbrot law

Here we maximize the Shannon entropy using the method of Lagrange multiplier under some equation constraints and get the Zipf–Mandelbrot law.

### Theorem 6.1

*If* $J = \{ 1,2, \ldots,N\}$, *for given* $q \ge 0$, *a probability distribution that maximizes the Shannon entropy under the constraints*

$$ \sum_{s \in J} r_{s} = 1,\qquad \sum _{s \in J} r_{s} \bigl( \ln (s + q) \bigr): = \varPsi $$

*is the Zipf–Madelbrot law*.

### Proof

If $J = \{ 1,2, \ldots,N\}$, we set the Lagrange multipliers *λ* and *t* and consider the expression

$$ \hat{S} = - \sum_{s = 1}^{N} r_{s}\ln r_{s} - \lambda \Biggl( \sum _{s = 1}^{N} r_{s} - 1 \Biggr) - t \Biggl( \sum_{s = 1}^{N} r_{s}\ln (s + q) - \varPsi \Biggr). $$

Just for the sake of convenience, replace *λ* by $\ln \lambda - 1$, thus the last expression gives

$$ \hat{S} = - \sum_{s = 1}^{N} r_{s}\ln r_{s} - ( \ln \lambda - 1 ) \Biggl( \sum _{s = 1}^{N} r_{s} - 1 \Biggr) - t \Biggl( \sum_{s = 1}^{N} r_{s}\ln (s + q) - \varPsi \Biggr). $$

From $\hat{S}_{r_{s}} = 0$, for $s = 1,2, \ldots,N$, we get

$$ r_{s} = \frac{1}{\lambda ( s + q ) ^{t}}, $$

and on using the constraint $\sum_{s = 1}^{N} r_{s} = 1$, we have

$$ \lambda = \sum_{s = 1}^{N} \biggl( \frac{1}{(s + 1)^{t}} \biggr), $$

where $t > 0$, concluding that

$$ r_{s} = \frac{1}{(s + q)^{t}H_{N,q,t}}, \quad s = 1,2, \ldots,N. $$

 □

### Remark 6.2

Observe that the Zipf–Mandelbrot law and Shannon entropy can be bounded from above (see [23]).

$$ S = - \sum_{s = 1}^{N} f ( s,N,q,t ) \ln f(s,N,q,t) \le - \sum_{s = 1}^{N} f(s,N,q,t) \ln q_{s}, $$

where $( q_{1}, \ldots,q_{N} ) $ is a positive *N*-tuple such that $\sum_{s = 1}^{N} q_{s} = 1$.

### Theorem 6.3

*If* $J = \{ 1, \ldots,N\}$, *then the probability distribution that maximizes Shannon entropy under constraints*

$$ \sum_{s \in J} r_{s} = 1,\qquad \sum _{s \in J} r_{s}\ln (s + q): = \varPsi,\qquad \sum_{s \in J} sr_{s}: = \eta $$

*is a hybrid Zipf–Mandelbrot law given as*

$$ r_{s} = \frac{w^{s}}{ ( s + q ) ^{k}\varPhi^{*}(k,q,w)},\quad s \in J, $$

*where*

$$ \varPhi_{J}(k,q,w) = \sum_{s \in J} \frac{w^{s}}{(s + q)^{k}}. $$

### Proof

First consider $J = \{ 1, \ldots,N\}$, we set the Lagrange multiplier and consider the expression

$$ \tilde{S} = - \sum_{s = 1}^{N} r_{s}\ln r_{s} + \ln w \Biggl( \sum _{s = 1} ^{N} sr_{s} - \eta \Biggr) - ( \ln \lambda - 1 ) \Biggl( \sum_{s = 1}^{N} r_{s} - 1 \Biggr) - k \Biggl( \sum_{s = 1}^{N} r_{s}\ln (s + q) - \varPsi \Biggr). $$

On setting $\tilde{S}_{r_{s}} = 0$, for $s = 1, \ldots,N$, we get

$$ - \ln r_{s} + s\ln w - \ln \lambda - k\ln(s + q) = 0. $$

After solving for $r_{s}$, we get

$$ \lambda = \sum_{s = 1}^{N} \frac{w^{s}}{ ( s + q ) ^{k}}, $$

and we recognize this as the partial sum of Lerch’s transcendent that we will denote by

$$ \varPhi_{N}^{ *} ( k,q,w ) = \sum _{s = 1}^{N} \frac{w^{s}}{(s + q)^{k}} $$

with $w \ge 0$, $k > 0$. □

### Remark 6.4

Observe that for the Zipf–Mandelbrot law, Shannon entropy can be bounded from above (see [23]).

$$ S = - \sum_{s = 1}^{N} f_{h} ( s,N,q,k ) \ln f_{h} ( s,N,q,k ) \le - \sum _{s = 1}^{N} f_{h} ( s,N,q,k ) \ln q_{s}, $$

where $( q_{1}, \ldots,q_{N} ) $ is any positive *N*-tuple such that $\sum_{s = 1}^{N} q_{s} = 1$.

Under the assumption of Theorem 2.1(i), define the nonnegative functionals as follows:

50 $$\begin{aligned}& \varTheta_{3}(f) = \mathsf{A}_{m,r}^{[1]} - f \biggl( \frac{\sum_{s = 1}^{n} r _{s}}{\sum_{s = 1}^{n} q_{s}} \biggr) \sum_{s = 1}^{n} q_{s},\quad r = 1, \ldots,m, \\ \end{aligned}$$

51 $$\begin{aligned}& \varTheta_{4}(f) = \mathsf{A}_{m,r}^{[1]} - \mathsf{A}_{m,k}^{[1]},\quad 1 \le r < k \le m. \end{aligned}$$

Under the assumption of Theorem 2.1(ii), define the nonnegative functionals as follows:

52 $$\begin{aligned}& \varTheta_{5}(f) = \mathsf{A}_{m,r}^{[2]} - \Biggl( \sum _{s = 1}^{n} r_{s} \Biggr) f \biggl( \frac{\sum_{s = 1}^{n} r_{s}}{\sum_{s = 1}^{n} q_{s}} \biggr),\quad r = 1, \ldots,m, \end{aligned}$$

53 $$\begin{aligned}& \varTheta_{6}(f) = \mathsf{A}_{m,r}^{[2]} - \mathsf{A}_{m,k}^{[2]}, \quad 1 \le r < k \le m. \end{aligned}$$

Under the assumption of Corollary 3.1(i), define the following nonnegative functionals:

54 $$\begin{aligned}& \varTheta_{7}(f) = A_{m,r}^{[3]} + \sum _{i = 1}^{n} q_{i}\log (q_{i}), \quad r = 1, \ldots,n, \end{aligned}$$

55 $$\begin{aligned}& \varTheta_{8}(f) = A_{m,r}^{[3]} - A_{m,k}^{[3]},\quad 1 \le r < k \le m. \end{aligned}$$

Under the assumption of Corollary 3.1(ii), define the following nonnegative functionals given as

56 $$\begin{aligned}& \varTheta_{9}(f) = A_{m,r}^{[4]} - S,\quad r = 1, \ldots,m, \end{aligned}$$

57 $$\begin{aligned}& \varTheta_{10}(f) = A_{m,r}^{[4]} - A_{m,k}^{[4]},\quad 1 \le r < k \le m. \end{aligned}$$

Under the assumption of Corollary 3.2(i), let us define the nonnegative functionals as follows:

58 $$\begin{aligned}& \varTheta_{11}(f) = A_{m,r}^{[5]} - \sum _{s = 1}^{n} r_{s}\log \Biggl( \sum _{s = 1}^{n} \log \frac{r_{n}}{\sum_{s = 1}^{n} q_{s}} \Biggr),\quad r = 1, \ldots,m, \end{aligned}$$

59 $$\begin{aligned}& \varTheta_{12}(f) = A_{m,r}^{[5]} - A_{m,k}^{[5]}, \quad 1 \le r < k \le m. \end{aligned}$$

Under the assumption of Corollary 3.2(ii), define the nonnegative functionals as follows:

60 $$ \varTheta_{13}(f) = A_{m,r}^{[6]} - A_{m,k}^{[6]},\quad 1 \le r < k \le m. $$

Under the assumption of Theorem 4.1(i), consider the following functionals:

61 $$\begin{aligned}& \varTheta_{14}(f) = A_{m,r}^{[7]} - D_{\lambda } (\mathbf{r}, \mathbf{q}),\quad r = 1, \ldots,m, \end{aligned}$$

62 $$\begin{aligned}& \varTheta_{15}(f) = A_{m,r}^{[7]} - A_{m,k}^{[7]},\quad 1 \le r < k \le m. \end{aligned}$$

Under the assumption of Theorem 4.1(ii), consider the following functionals:

63 $$\begin{aligned}& \varTheta_{16}(f) = A_{m,r}^{[8]} - D_{1}(\mathbf{r},\mathbf{q}),\quad r = 1, \ldots,m, \\ \end{aligned}$$

64 $$\begin{aligned}& \varTheta_{17}(f) = A_{m,r}^{[8]} - A_{m,k}^{[8]}, \quad 1 \le r < k \le m. \end{aligned}$$

Under the assumption of Theorem 4.1(iii), consider the following functionals:

65 $$\begin{aligned}& \varTheta_{18}(f) = A_{m,r}^{[9]} - D_{\lambda } (\mathbf{r}, \mathbf{q}),\quad r = 1, \ldots,m, \end{aligned}$$

66 $$\begin{aligned}& \varTheta_{19}(f) = A_{m,r}^{[9]} - A_{m,k}^{[9]},\quad 1 \le r < k \le m. \end{aligned}$$

Under the assumption of Theorem 4.2, consider the following nonnegative functionals:

67 $$\begin{aligned}& \varTheta_{20}(f) = D_{\lambda } (\mathbf{r},\mathbf{q}) - A_{m,r} ^{[10]},\quad r = 1, \ldots,m, \end{aligned}$$

68 $$\begin{aligned}& \varTheta_{21}(f) = A_{m,k}^{[10]} - A_{m,r}^{[10]}, \quad 1 \le r < k \le m. \end{aligned}$$

69 $$\begin{aligned}& \varTheta_{22}(f) = A_{m,r}^{[11]} - D_{\lambda } (\mathbf{r}, \mathbf{q}),\quad r = 1, \ldots,m, \end{aligned}$$

70 $$\begin{aligned}& \varTheta_{23}(f) = A_{m,r}^{[11]} - A_{m,r}^{[11]},\quad 1 \le r < k \le m, \end{aligned}$$

71 $$\begin{aligned}& \varTheta_{24}(f) = A_{m,r}^{[11]} - A_{m,k}^{[10]},\quad r = 1, \ldots,m, k = 1, \ldots,m. \end{aligned}$$

Under the assumption of Corollary 4.3(i), consider the following nonnegative functionals:

72 $$\begin{aligned}& \varTheta_{25}(f) = H_{\lambda } (r) - A_{m,r}^{[12]}, \quad r = 1, \ldots,m, \end{aligned}$$

73 $$\begin{aligned}& \varTheta_{26}(f) = A_{m,k}^{[12]} - A_{m,r}^{[12]},\quad 1 \le r < k \le m. \end{aligned}$$

Under the assumption of Corollary 4.3(ii), consider the following functionals:

74 $$\begin{aligned}& \varTheta_{27}(f) = S - A_{m,r}^{[13]}, \quad r = 1, \ldots,m, \end{aligned}$$

75 $$\begin{aligned}& \varTheta_{28}(f) = A_{m,k}^{[13]} - A_{m,r}^{[13]},\quad 1 \le r < k \le m. \end{aligned}$$

Under the assumption of Corollary 4.3(iii), consider the following functionals:

76 $$\begin{aligned}& \varTheta_{29}(f) = H_{\lambda } (\mathbf{r}) - A_{m,r}^{[14]},\quad r = 1, \ldots,m, \end{aligned}$$

77 $$\begin{aligned}& \varTheta_{30}(f) = A_{m,k}^{[14]} - A_{m,r}^{[14]},\quad 1 \le r < k \le m. \end{aligned}$$

Under the assumption of Corollary 4.4, define the following functionals:

78 $$\begin{aligned}& \varTheta_{31} = A_{m,r}^{[15]} - H_{\lambda } (r),\quad r = 1, \ldots,m, \end{aligned}$$

79 $$\begin{aligned}& \varTheta_{32} = A_{m,r}^{[15]} - A_{m,k}^{[15]},\quad 1 \le r < k \le m, \end{aligned}$$

80 $$\begin{aligned}& \varTheta_{33} = H_{\lambda } (\mathbf{r}) - A_{m,r}^{[16]}, \quad r = 1, \ldots,m, \end{aligned}$$

81 $$\begin{aligned}& \varTheta_{34} = A_{m,k}^{[16]} - A_{m,r}^{[16]},\quad 1 \le r < k \le m, \end{aligned}$$

82 $$\begin{aligned}& \varTheta_{35} = A_{m,r}^{[15]} - A_{m,k}^{[16]},\quad r = 1, \ldots,m, k = 1, \ldots,m. \end{aligned}$$

## Generalization of the refinement of Jensen’s, Rényi, and Shannon type inequalities via Montgomery identity

We construct some new identities with the help of the generalized Montgomery identity (5).

### Theorem 7.1

*Assume* ($H_{1}$), *let* $f:[\alpha_{1},\alpha_{2}] \to \mathsf{R}$ *be a function where* $[\alpha_{1}, \alpha_{2}] \subset \mathsf{R}$ *is an interval*. *Also let* $x_{1}, \ldots,x_{n} \in [\alpha_{1},\alpha_{2}]$ *and* $p_{1}, \ldots,p_{n}$ *be positive real numbers such that* $\sum_{i = 1}^{n} p_{i} = 1$, *and* $R_{m}(x,u)$ *be the same as defined in* (6), *then the following identity holds*:

83 $$\begin{aligned} \varTheta_{i}(f) =& \frac{1}{\alpha_{2} - \alpha_{1}}\sum _{k = 0}^{m - 2} \biggl( \frac{1}{k!(k + 2)} \biggr) \bigl( f^{(k + 1)}(\alpha_{1})\varTheta _{i} \bigl((x - \alpha_{1})^{k + 1} \bigr) - f^{(k + 1)}( \alpha_{2}) \\ &{} \times \varTheta_{2} \bigl((x - \alpha_{2})^{k + 1} \bigr) \bigr) \frac{1}{(m - 1)!} \int_{\alpha_{1}}^{\alpha_{2}} \varTheta_{i} \bigl(R_{m}(x,u) \bigr)f^{(m)}(u)\,du,\quad i = 1, \ldots, 35. \end{aligned}$$

### Proof

Using (5) in (2), (3), and (50)–(82), we get the result. □

### Theorem 7.2

*Assume* ($H_{1}$), *let* $f:[\alpha_{1},\alpha_{2}] \to \mathsf{R}$ *be a function where* $[\alpha_{1}, \alpha_{2}] \subset \mathsf{R}$ *is an interval*. *Also let* $x_{1}, \ldots,x_{n} \in [\alpha_{1},\alpha_{2}]$ *and* $p_{1}, \ldots,p_{n}$ *be positive real numbers such that* $\sum_{i = 1}^{n} p_{i} = 1$, *and* $R_{m}(x,u)$ *be the same as defined in* (6). *Let*, *for* $m \ge 2$,

$$ \varTheta_{i} \bigl(R_{m}(x,u) \bigr) \ge 0 \quad \textit{for all }u \in [\alpha_{1},\alpha_{2}], i = 1, \ldots,35. $$

*If* *f* *is* *m*-*convex such that* $f^{(m - 1)}$ *is absolutely continuous*, *then*

84 $$\begin{aligned} \varTheta_{i}(f) \ge& \frac{1}{\alpha_{2} - \alpha_{1}}\sum _{k = 0}^{m - 2} \biggl( \frac{1}{k!(k + 2)} \biggr) \\ & {}\times ( f^{(k + 1)}(\alpha_{1})\varTheta _{i} \bigl((x - \alpha_{1})^{k + 1} \bigr)- f^{(k + 1)}( \alpha_{2})\varTheta_{i} \bigl((x - \alpha_{2})^{k + 1} \bigr) ,\quad i = 1, \ldots,35. \end{aligned}$$

### Proof

As $f^{(m - 1)}$ is absolutely continuous on $[\alpha_{1},\alpha_{2}]$, therefore $f^{(m)}$ exists almost everywhere. As *f* is *m*-convex, so $f^{(m)}(u) \ge 0$ for all $u \in [\alpha_{1},\alpha_{2}]$ (see [25, p. 16]). Hence, using Theorem 7.1, we get (84). □

### Theorem 7.3

*Assume* ($H_{1}$), *let* $f:[\alpha_{1},\alpha_{2}] \to \mathsf{R}$ *be a function where* $[\alpha_{1}, \alpha_{2}] \subset \mathsf{R}$ *is an interval*. *Also let* $x_{1}, \ldots,x_{n} \in [\alpha_{1},\alpha_{2}]$ *and* $p_{1}, \ldots,p_{n}$ *be positive real numbers such that* $\sum_{i = 1}^{n} p_{i} = 1$, *let* $f:[\alpha_{1},\alpha_{2}] \to \mathsf{R}$ *be a convex function*.

(i) *If* $m \ge 2$ *is even*, *then* (84) *holds*.
(ii) *Let* (84) *be valid*. *If the function*
  $$ \lambda (x) = \frac{1}{\alpha_{2} - \alpha_{1}}\sum_{l = 0}^{m - 2} \biggl( \frac{f^{(l + 1)}(\alpha_{1})(x - \alpha_{1})^{l + 2} - f^{(l + 1)}(\alpha_{2})(x - \alpha_{2})^{l + 2}}{l!(l + 2)} \biggr) $$

*is convex*, *then the right*-*hand side of* (84) *is nonnegative and*

$$ \varTheta_{i}(f) \ge 0,\quad i = 1, \ldots,35. $$

### Proof

(i) The function $R_{m}( \cdot,v)$ is convex (see [10]). Hence, for an even integer $m \ge 2$,
  $$ \varTheta_{i} \bigl(R_{m}(u,v) \bigr) \ge 0, $$
  therefore from Theorem 7.2, we have (84).
(ii) By using the linearity of $\varTheta_{i}(f)$, we can write the right-hand side of (84) in the form $\varTheta_{i}(\lambda)$. As *λ* is supposed to be convex, therefore the right-hand side of (84) is nonnegative, so $\varTheta_{i}(f) \ge 0$.

 □

### Theorem 7.4

*Assume* ($H_{1}$), *let* $f:[ \alpha_{1},\alpha_{2}] \to \mathsf{R}$ *be a function where* $[\alpha_{1},\alpha_{2}] \subset \mathsf{R}$ *is an interval*. *Also let* $x_{1}, \ldots,x_{n} \in [\alpha_{1},\alpha_{2}]$ *and* $p_{1}, \ldots,p_{n}$ *be positive real numbers such that* $\sum_{i = 1}^{n} p_{i} = 1$, *and* $\hat{R}_{m}(x,u)$ *be the same as defined in* (8), *then the following identity holds*:

85 $$\begin{aligned} \varTheta_{i}(f) =& \frac{1}{\alpha_{2} - \alpha_{1}}\sum _{k = 0}^{m - 2} \biggl( \frac{1}{k!(k + 2)} \biggr) ( \varTheta_{i} \bigl(f^{(k + 1)}(x) ( \alpha_{1} - x)^{k + 1} \bigr) - \varTheta_{i} \bigl(f^{(k + 1)}(x) ( \alpha_{2} - x)^{k + 1} \bigr) \\ &{}+ \frac{1}{(m - 1)!} \int_{\alpha_{1}}^{\alpha_{2}} \varTheta_{i} \bigl( \hat{R}_{m}(x,u) \bigr)f^{(m)}(u)\,du,\quad i = 1, \ldots,35. \end{aligned}$$

### Proof

Using (7) in (2), (3), and (50)–(82), we get identity (85). □

### Theorem 7.5

*Assume* ($H_{1}$), *let* $f:[ \alpha_{1},\alpha_{2}] \to \mathsf{R}$ *be a function where* $[\alpha_{1},\alpha_{2}] \subset \mathsf{R}$ *is an interval*. *Also let* $x_{1}, \ldots,x_{n} \in [\alpha_{1},\alpha_{2}]$ *and* $p_{1}, \ldots,p_{n}$ *be positive real numbers such that* $\sum_{i = 1}^{n} p_{i} = 1$, *and* $\hat{R}_{m}(x,u)$ *be the same as defined in* (8). *Let*, *for* $m \ge 2$,

$$ \varTheta_{i} \bigl(\hat{R}_{m}(x,u) \bigr) \ge 0\quad \textit{for all }u \in [\alpha_{1},\alpha_{2}], i = 1, \ldots,35. $$

*If* *f* *is* *m*-*convex such that* $f^{(m - 1)}$ *is absolutely continuous*, *then*

86 $$\begin{aligned} \varTheta_{i}(f) \ge& \frac{1}{\alpha_{2} - \alpha_{1}}\sum _{k = 0}^{m - 2} \biggl( \frac{1}{k!(k + 2)} \biggr) \\ &{}\times ( \varTheta_{i} \bigl(f^{(k + 1)}(x) ( \alpha_{1} - x)^{k + 1} \bigr) - \varTheta_{i} \bigl(f^{(k + 1)}(x) (\alpha_{2} - x)^{k + 1} \bigr),\quad i = 1, \ldots,35. \end{aligned}$$

### Proof

As $f^{(m - 1)}$ is absolutely continuous on $[\alpha _{1},\alpha_{2}]$, therefore $f^{(m)}$ exists almost everywhere. As *f* is *m*-convex, so $f^{(m)}(u) \ge 0$ for all $u \in [\alpha_{1}, \alpha_{2}]$ (see [25, p. 16]). Hence, using Theorem 7.4, we get (86). □

### Remark 7.6

We can get a similar result as that given in Theorem 7.3.

### Remark 7.7

We can give related mean value theorems, also construct the new families of *m*-exponentialy convex functions and Cauchy means related to the functionals $\varTheta_{i}$, $i = 1, \ldots,35$, as given in [7].
